# Supplementary material for: Deciphering tumour tissue organization by 3D electron microscopy and machine learning
Source: Commun Biol. 2021 Dec 13;4:1390. doi: 10.1038/s42003-021-02919-z (PMC8668903; doi:10.1038/s42003-021-02919-z)
Supplement: Supplementary file 1 — Supplementary Information [file 42003_2021_2919_MOESM1_ESM.pdf]

a

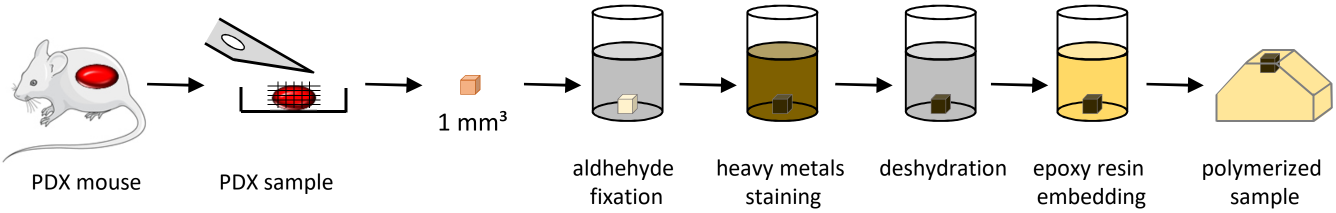

b

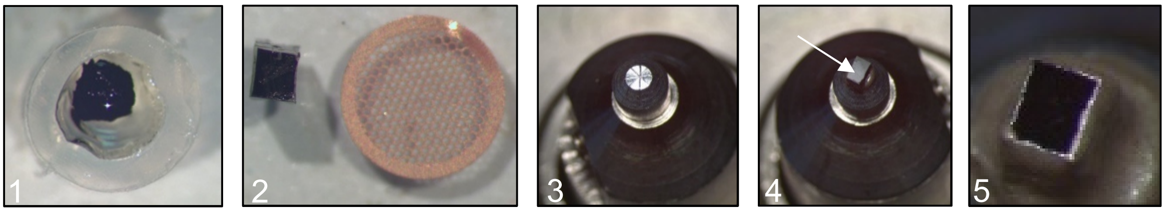

Supplementary Fig. 1: SBF-SEM Sample preparation workflow. (**a**) Serial block-face (SBF) chemical sample preparation steps from HB-PDX tumour fragment extraction to sample resin embedding. (**b**) Mounting of block on SBF-SEM pin. 1: Sample in resin block, 2: Block after trimming; 3: SBF pin; 4: Sample block mounted (white arrow) on SBF pin; 5: Flat block area ready for acquisition.

Sample 1

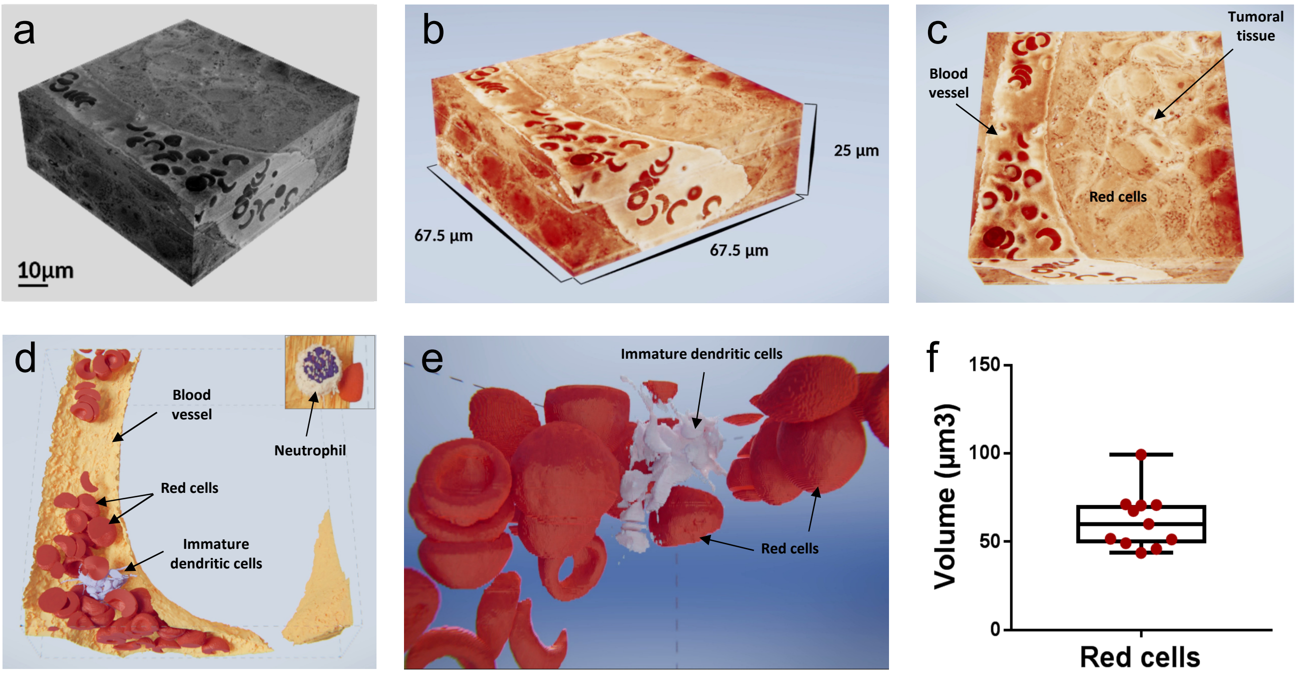

Supplementary Fig. 2. Digital representation of blood capillary portions and blood cells from HB PDX Sample 1. (**a**) Stack of 250 images after 3D volume reconstruction. (**b**) Digital representation of stack in A with dimensions of block in µm on X-, Y- and Z- axes. (**c**) Top view of digital stack with biological annotations. (**d**) Top view of capillary portions and circulating murine blood cells with biological annotations. Top right insert: neutrophil circulating in capillary portion located in right bottom corner of stack. (**e**) Digital representation of circulating red cells and immature dendritic cells. (**f**) Graphical representation of volumetric distribution of 11 fully contained red cells (Mean +/- Standard Deviation = 61.92 +/- 16.26 µm^3; Median = 60.03 µm^3; Minimum = 43.64 µm^3; Maximum = 99.37 µm^3).

Sample 2

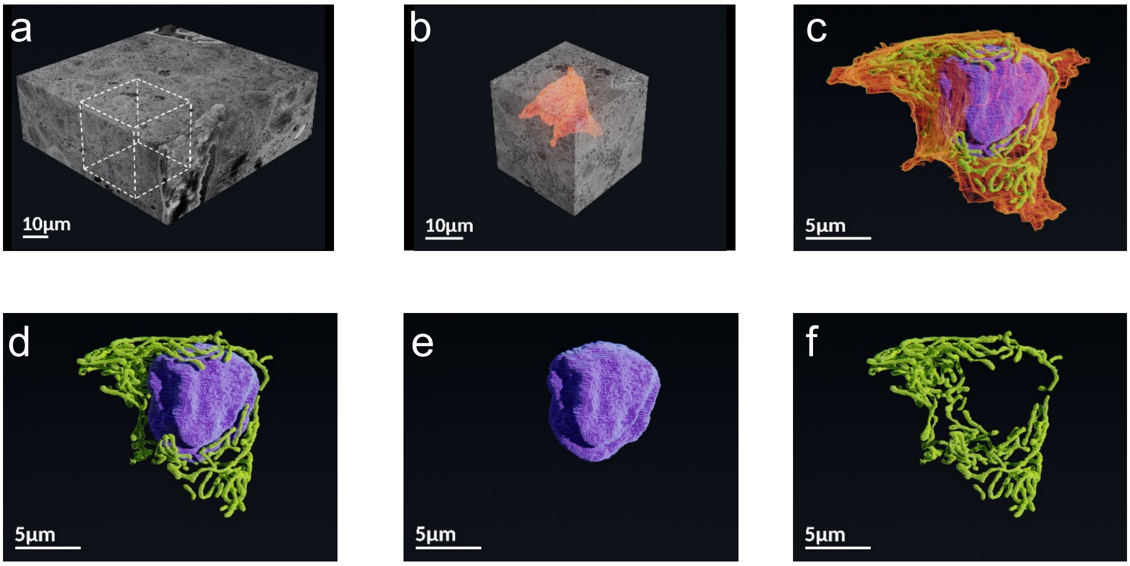

Supplementary Fig. 3. Digital representation of a tumour cell from HB PDX Sample 2. (**a**) Stack of 246 images after 3D volume reconstruction. White dotted-line box shows region-of-interest (ROI). (**b**) ROI containing analysed tumour cell. (**c-f**) Digital representations of tumour cell (V = 970.6 μm^3) and its cytoplasm in orange (**c**, V = 657.1 μm^3), its nucleus in blue (**d** and **e**, V = 313.6 μm^3) and its mitochondrial network in green (**d** and **f**, V = 64.6 μm^3).

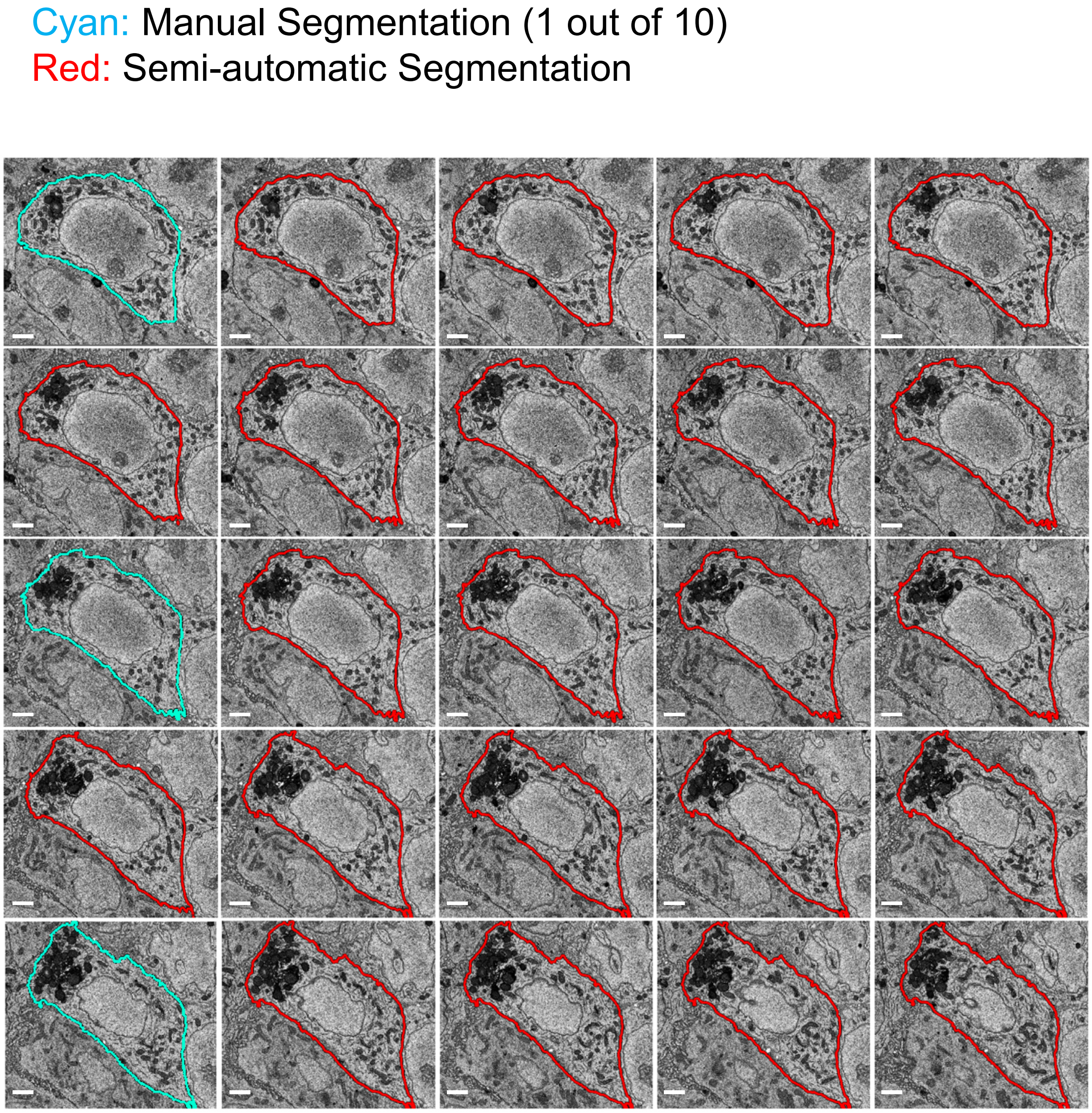

Supplementary Fig. 4. Example of semi-automatic segmentation method used to segment cells. Cyan lines: region-of-interest (ROI) encompassing a cell defined by hand on 1 out of 10 2D images along Z-axis. Red lines: manually defined ROIs propagated to neighbouring slices using a so-called « Optical Flow » algorithm applied on acquired EM images. Scale bar = 2 µm.

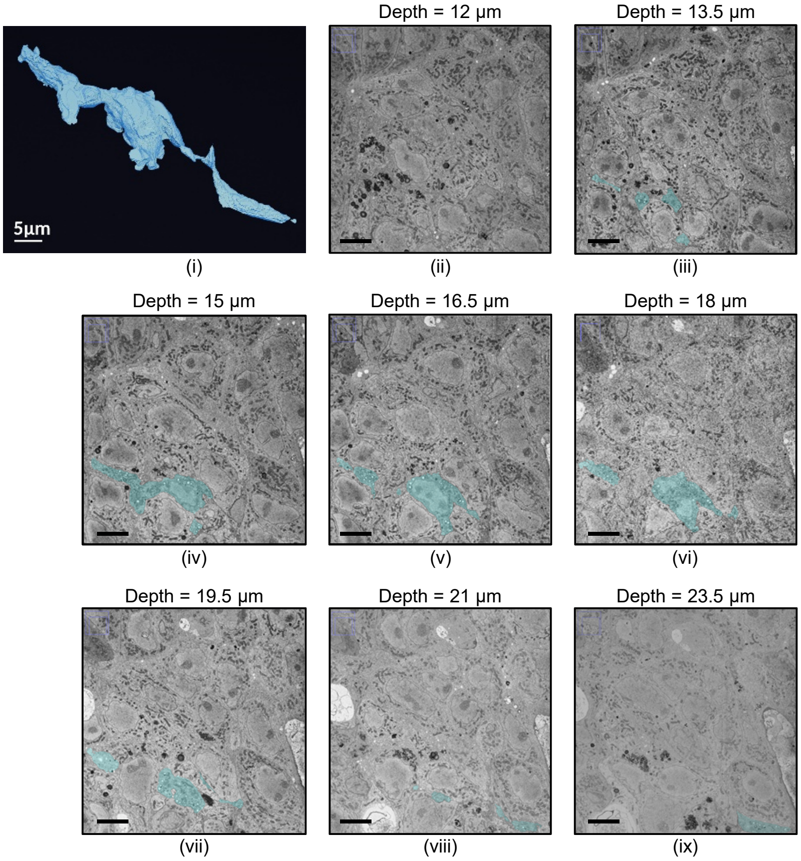

Supplementary Fig. 5. Immune cells from HB PDX sample 2. (i) 3D digital representation in cyan of a tumour tissue infiltrating immune cell, likely a monocyte/macrophage. (ii-ix) 2D cross-section maps are reported for increasing depth along Z-axis for segmented portions of immune cell. Scale bar = 10 µm.

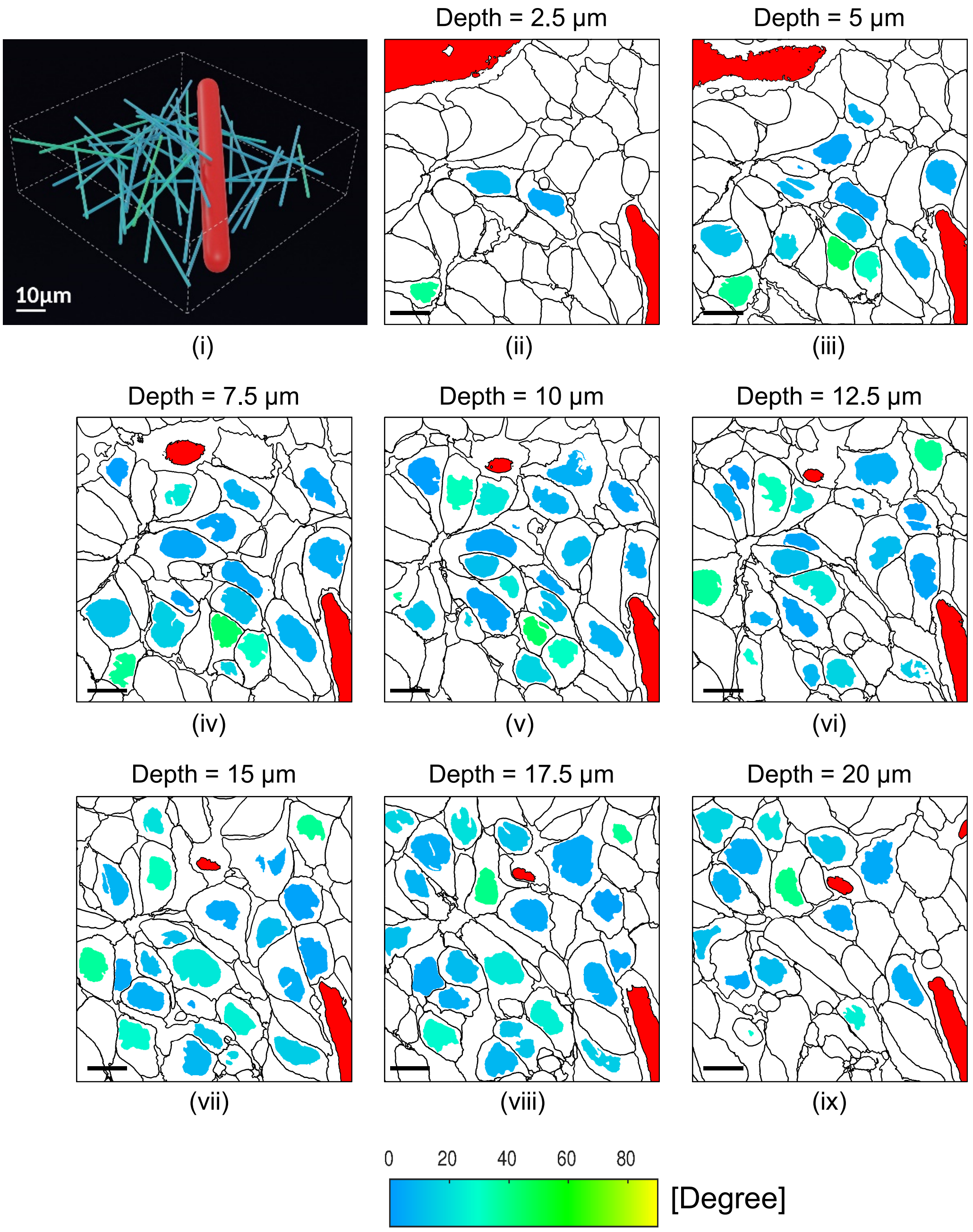

Supplementary Fig. 6. Analysis of tumour cell nuclei and blood capillary alignment. (i) Main axis of each structure is represented as a stick (colour sticks = segmented complete nuclei, red stick = blood capillary). Major axes of nuclei were used to calculate best alignment plane (least square sense); (ii-ix) Angles calculated in degrees (see colour scale) between main axis of each nucleus and alignment plane. 2D cross-section maps are reported for increasing depth along Z-axis (i.e., direction of cut through sample). Blood capillary portions are shown in red. Scale bar = 10 µm.

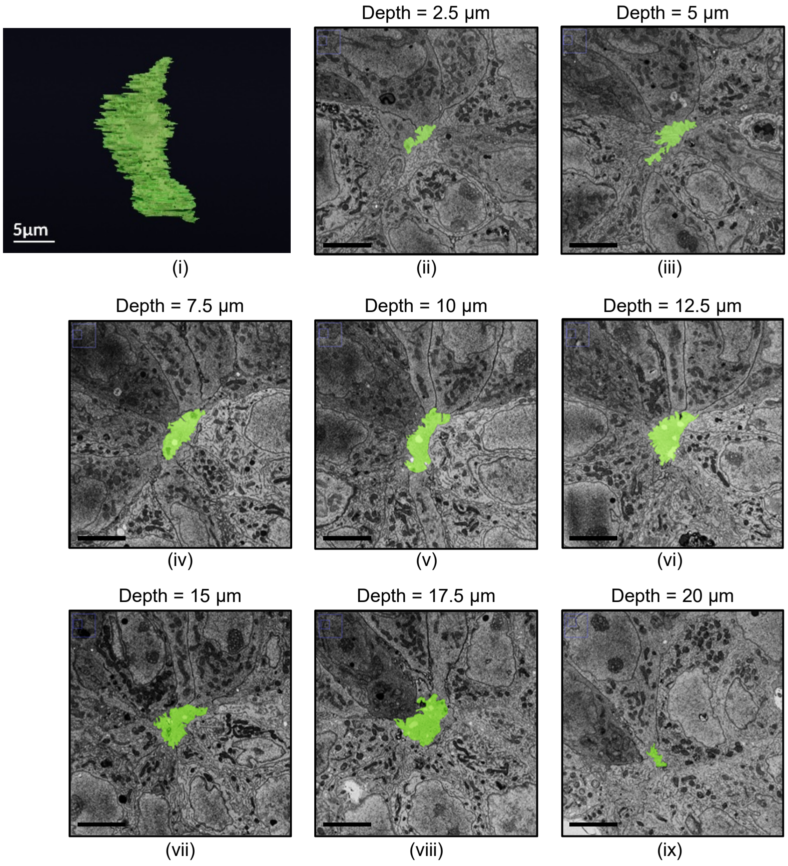

Supplementary Fig. 7. SBF-SEM images of bile canaliculus-like structure from Sample 2. (i) 3D digital representation in green of a bile canaliculus-like structure. (ii-ix) 2D cross-sectional maps are reported for increasing depth along Z-axis for segmented portions of bile canaliculus-like structure. Scale bar = 5 μm.

a

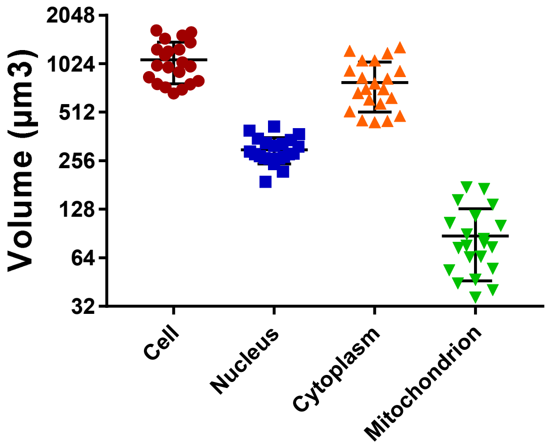

	Cell	Nucleus	Cytoplasm	Mitochondrion
Number of values	21	21	21	21
Minimum	670.2	190	443	36.31
25% Percentile	785	265.3	549.5	54.21
Median	997.1	283.8	715.4	76.1
75% Percentile	1322	337.2	1001	110.8
Maximum	1647	417.7	1295	175.2
Mean	1082	300.1	782.9	87.31
Std. Deviation	312.3	55.95	269.3	41.23
Std. Error of Mean	68.15	12.21	58.76	8.998

b

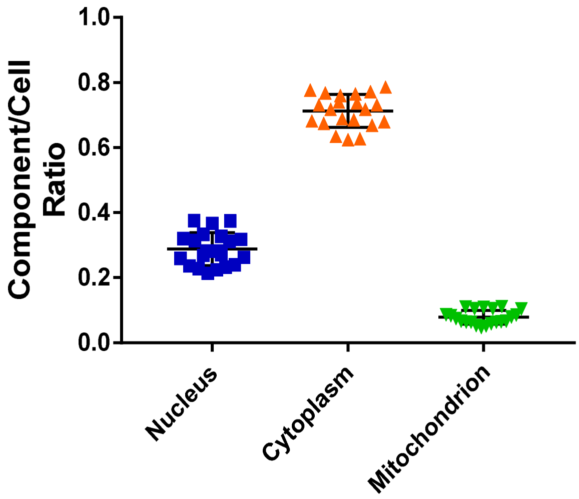

	Nucleus/cell ratio	Cytoplasm/cell ratio	Mitochondrial network/cell ratio
Number of values	21	21	21
Minimum	0.2381	0.6771	0.06294
25% Percentile	0.2821	0.7179	0.07456
Median	0.324	0.7629	0.1041
75% Percentile	0.3754	0.7862	0.1114
Maximum	0.2138	0.6246	0.04733
Mean	0.2877	0.7129	0.07853
Std. Deviation	0.05074	0.05057	0.02134
Std. Error of Mean	0.01107	0.01104	0.004658

Supplementary Fig. 8. Quantitative measurements of tumour cells and their subcellular components. (**a**) Graph shows volumetric sizes of 21 fully contained cells and their cytoplasm, nuclei and mitochondrial networks. (**b**) Graph shows corresponding component/cell ratios. (**a-b**) For each graph, detailed statistical data are shown in table on right.

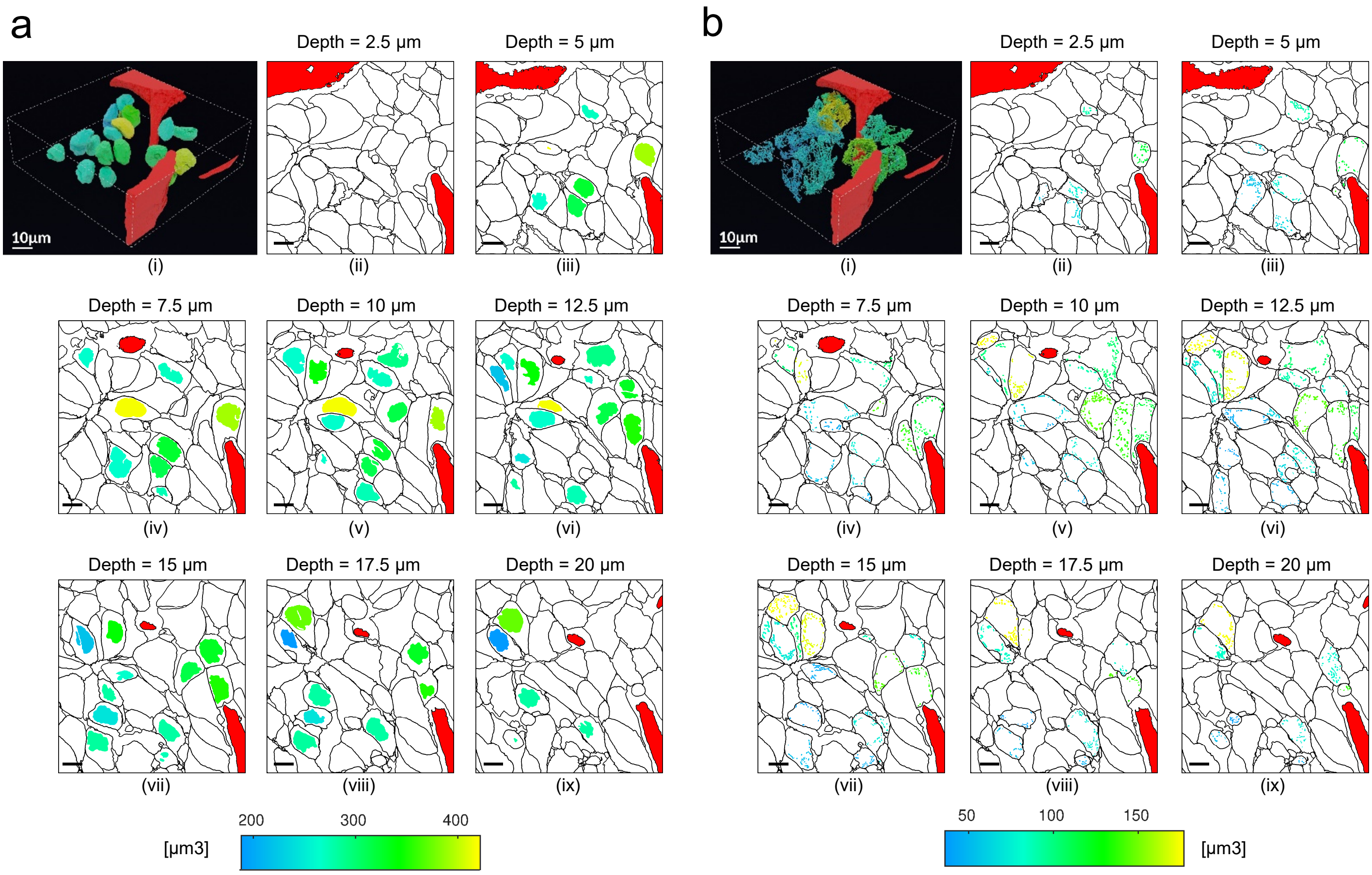

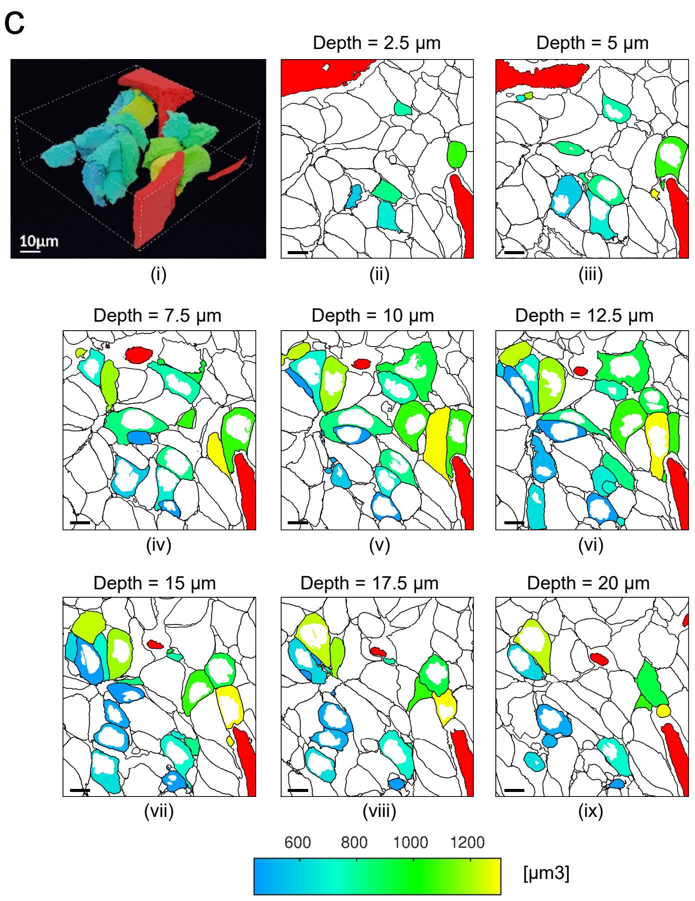

Supplementary Fig. 9. Relationship between distance to blood capillary and size of cellular components. Note that only 21 fully contained components in image field-of-view are considered here. (**a-c**) Visualization of sizes related to distance to blood capillary for a given component (**a**, nucleus; **b**, mitochondrial network; **c**, cytoplasm): (i) Reconstructed 3D image of corresponding component and blood capillary. Colour of a given component is reported according to its volumetric size (see the corresponding colour scale). (ii-ix) 2D cross-sectional maps are reported for increasing depth along Z-axis for corresponding component. Blood capillary portions are shown in red. Scale bar = 10 µm.

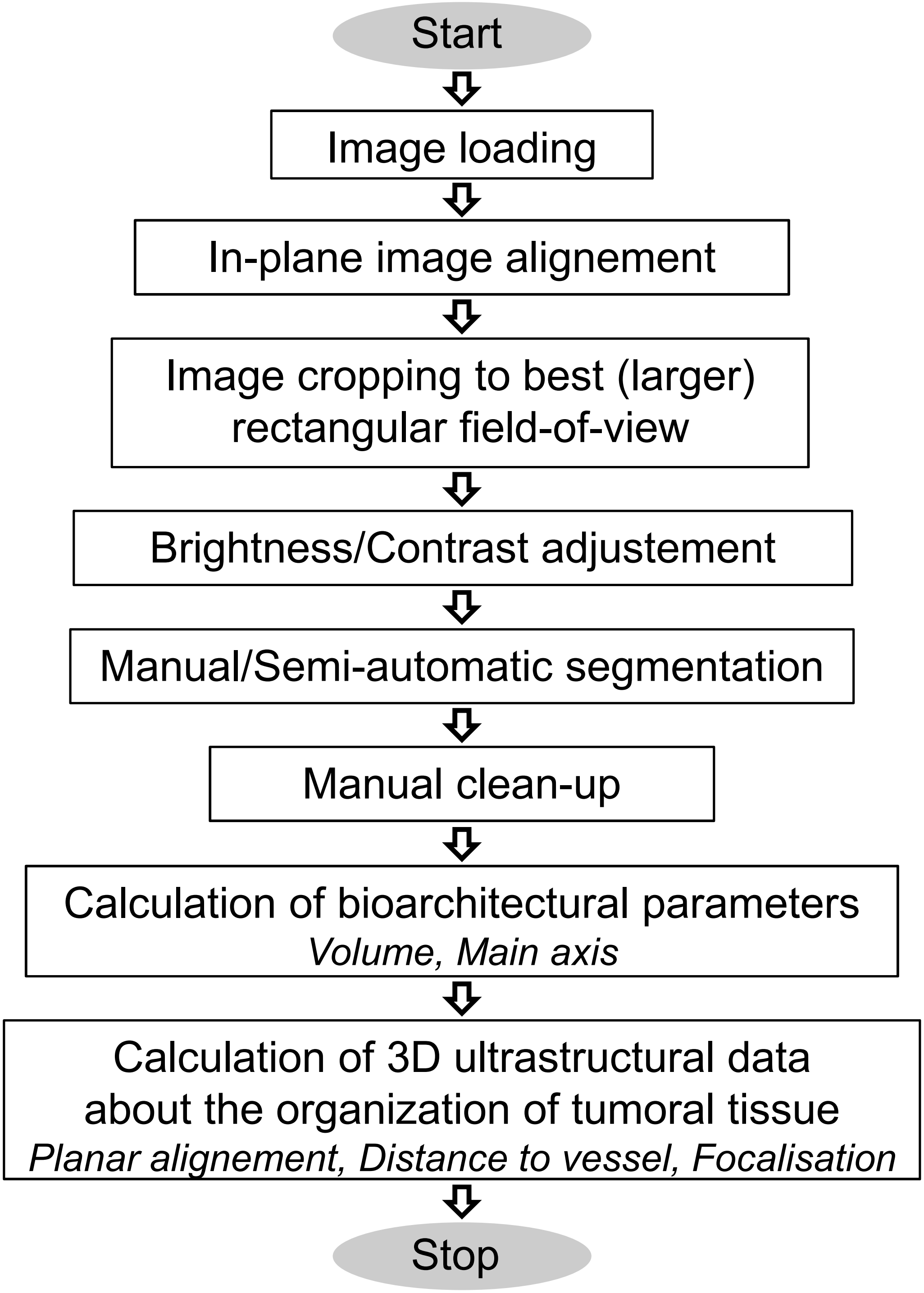

Supplementary Fig. 10. Pipeline of image processing.

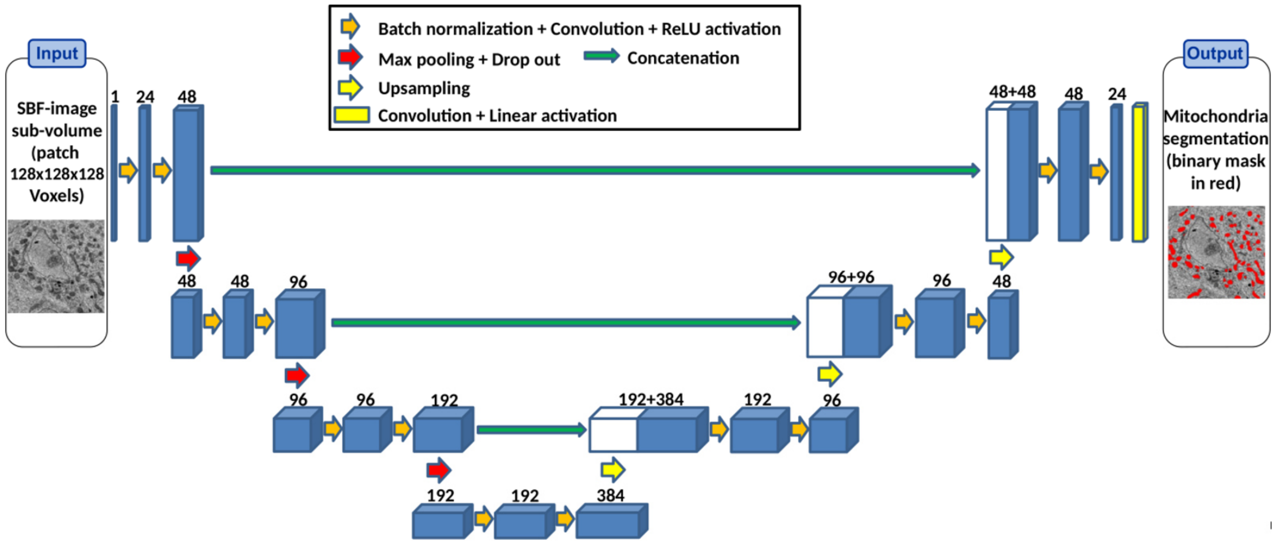

Supplementary Fig.11: Illustration of the used U-Net architecture for the segmentation of mitochondria. Due to limited Graphics Processing Unit (GPU) memory, we used a patch-wise strategy: local image areas (patch size=128×128×128 voxels) in the serial block-face images are used as a single input channel. Each block of the CNN (blue rectangle) is composed of batch normalization, convolution and ReLU activation. The number of 3x3x3 filters is indicated on the top of each block.

Supplementary Table 1: Main clinical and genetic characteristics of HB PDX samples

	Sample 1	Sample 2	Sample 3	Sample 4
**PDX ID**	HB-316	HB-319	HB-312	HB-310
**Age (months) > 3 years**	Yes	No	Yes	No
**Type of sample**	Primary tumour	Primary tumour	Intrahepatic recurrence	Primary tumour
**Sex**	Female	Female	Female	Female
**Vascular invasion**	Yes	No	ND	Yes
**Solitary/Multiple nodules**	M	ND	ND	M
**Metastasis**	Yes	No	ND	No
**Histology**	Epithelial	Mixed	Epithelial	Mixed
**Main epithelial component**	Embryonal, Macro-trabecular	Embryonal, Macro-trabecular	Foetal	Embryonal
**Small cells presence**	ND	No	No	No
**PRETEXT stage**	III	ND	ND	III
**Chemotherapy**	Yes	Yes	Yes	Yes
**High/Standard risk**	High	High	ND	High
**Serum AFP at diagnosis > 500,000 ng/mL**	No	No	ND	ND

ND: not determined.
