# Supplementary material for: Deciphering tumour tissue organization by 3D electron microscopy and machine learning
Source: Commun Biol. 2021 Dec 13;4:1390. doi: 10.1038/s42003-021-02919-z (PMC8668903; doi:10.1038/s42003-021-02919-z)
Supplement: Supplementary file 2 — Description of Additional Supplementary Files [file 42003_2021_2919_MOESM2_ESM.pdf]

Description of Additional Supplementary Files

File name: Supplementary Video 1.
Description: Spatial organization of blood capillary portions and circulating cells in HB PDX Sample 1. See also legend of Supplementary Fig.2.

File name: Supplementary Video 2.
Description: 3D organisation of tumour cell and its main subcellular components in HB PDX Sample 2. See legend of Supplementary Fig.3.

File name: Supplementary Video 3.
Description: Tumour and non-tumour components of HB PDX Sample 2. See legend of Fig. 2A-C.

File name: Supplementary Video 4.
Description: Alignment in plane of tumour cells or tumour cell nuclei versus blood capillary in HB PDX Sample 2. See legend of Fig. 2D.

File name: Supplementary Video 5.
Description: A set of tumour cells polarize in direction of a bile canaliculus-like structure. See legend of Fig. 3.

File name: Supplementary Video 6.
Description: Spatial organization of 21 cells and their organelles in HB PDX Sample 2. See legends of Fig. 4B and of Supplementary Fig. 9.

File name: Supplementary Data 1.
Description: File containing all values measured in our study and used to elaborate the Figure 2e, Figures 4a and 4c, Supplementary Figure 2f and Supplementary Figure 8a-b.
