# Supplementary material for: Deciphering tumour tissue organization by 3D electron microscopy and machine learning
Source: Commun Biol. 2021 Dec 13;4:1390. doi: 10.1038/s42003-021-02919-z (PMC8668903; doi:10.1038/s42003-021-02919-z)
Supplement: Supplementary file 10 — Reporting Summary [file 42003_2021_2919_MOESM10_ESM.pdf]

nature research

Corresponding author(s): Christophe F. Grosset
COMMSBIO-21-2178-T

Last updated by author(s): Oct 21, 2021

Reporting Summary

Nature Research wishes to improve the reproducibility of the work that we publish. This form provides structure for consistency and transparency in reporting. For further information on Nature Research policies, see our Editorial Policies and the Editorial Policy Checklist.

Statistics

For all statistical analyses, confirm that the following items are present in the figure legend, table legend, main text, or Methods section.

n/a	Confirmed	
☐	☒	The exact sample size (n) for each experimental group/condition, given as a discrete number and unit of measurement
☐	☒	A statement on whether measurements were taken from distinct samples or whether the same sample was measured repeatedly
☐	☒	The statistical test(s) used AND whether they are one- or two-sided<br>*Only common tests should be described solely by name; describe more complex techniques in the Methods section.*
☒	☐	A description of all covariates tested
☒	☐	A description of any assumptions or corrections, such as tests of normality and adjustment for multiple comparisons
☒	☐	A full description of the statistical parameters including central tendency (e.g. means) or other basic estimates (e.g. regression coefficient) AND variation (e.g. standard deviation) or associated estimates of uncertainty (e.g. confidence intervals)
☒	☐	For null hypothesis testing, the test statistic (e.g. F, t, r) with confidence intervals, effect sizes, degrees of freedom and P value noted<br>*Give P values as exact values whenever suitable.*
☒	☐	For Bayesian analysis, information on the choice of priors and Markov chain Monte Carlo settings
☒	☐	For hierarchical and complex designs, identification of the appropriate level for tests and full reporting of outcomes
☒	☐	Estimates of effect sizes (e.g. Cohen's d, Pearson's r), indicating how they were calculated

Our web collection on statistics for biologists contains articles on many of the points above.

Software and code

Policy information about availability of computer code

Data collection	Digital Micrograph (Gatan Inc., Pleasanton, CA, USA) was used to collect images acquired with a Hitachi H7650 transmission electron microscope (Japan).
Data analysis	Digital Micrograph (Gatan Inc., Pleasanton, CA, USA) was used to align the images with the Image Alignment plugin. Fiji and itksnap softwares were used to visualize/convert image/segmentations files. VAST-Lite software was used for the manual segmentation of the elements of interest (cells, nuclei and mitochondria). Manual segmentation was done using an interactive drawing tablet and pen (Cintiq pro 5, Wacom). A deep-learning algorithm was used to segment images using a 2D U-net architecture and an implementation based on Tensorflow 1.4 and Keras 2.2.4. Bioarchitectural parameters J were calculated using in-house algorithm developed under the commercial software Matlab ©1994-2021 The MathWorks, Inc.

For manuscripts utilizing custom algorithms or software that are central to the research but not yet described in published literature, software must be made available to editors and reviewers. We strongly encourage code deposition in a community repository (e.g. GitHub). See the Nature Research guidelines for submitting code & software for further information.

Data

Policy information about availability of data

All manuscripts must include a data availability statement. This statement should provide the following information, where applicable:

- Accession codes, unique identifiers, or web links for publicly available datasets
- A list of figures that have associated raw data
- A description of any restrictions on data availability

All data are available in the revised version of the manuscript.

Field-specific reporting

Please select the one below that is the best fit for your research. If you are not sure, read the appropriate sections before making your selection.

☒ Life sciences ☐ Behavioural & social sciences ☐ Ecological, evolutionary & environmental sciences

For a reference copy of the document with all sections, see nature.com/documents/nr-reporting-summary-flat.pdf

Life sciences study design

All studies must disclose on these points even when the disclosure is negative.

Sample size	We used 4 samples for electron microscopy imaging. One sample was manually segmented for some specific structures (cell, nucleus, mitochondria, blood capillary). Only one tumour tissue sample was fully investigated using 3D imaging, deep learning and mathematical algorythms.
Data exclusions	No data were excluded from the analyses.
Replication	No data were replicated as we used a very limited number of tumour tissue samples
Randomization	Not relevant for our study as we used a very limited number of tumour tissue samples
Blinding	Not relevant for our study as we used a very limited number of tumour tissue samples

Reporting for specific materials, systems and methods

We require information from authors about some types of materials, experimental systems and methods used in many studies. Here, indicate whether each material, system or method listed is relevant to your study. If you are not sure if a list item applies to your research, read the appropriate section before selecting a response.

Materials & experimental systems

n/a	Involved in the study	
☒	☐	Antibodies
☒	☐	Eukaryotic cell lines
☒	☐	Palaeontology and archaeology
☒	☐	Animals and other organisms
☒	☐	Human research participants
☒	☐	Clinical data
☒	☐	Dual use research of concern

Methods

n/a	Involved in the study	
☒	☐	ChIP-seq
☒	☐	Flow cytometry
☒	☐	MRI-based neuroimaging
